# Replicability and Reproducibility in Comparative Psychology

**DOI:** 10.3389/fpsyg.2017.00862

**Published:** 2017-05-26

**Authors:** Jeffrey R. Stevens

**Affiliations:** Department of Psychology and Center for Brain, Biology and Behavior, University of Nebraska-LincolnLincoln, NE, United States

**Keywords:** animal research, comparative psychology, pre-registration, replication, reproducible research

Psychology faces a replication crisis. The Reproducibility Project: Psychology sought to replicate the effects of 100 psychology studies. Though 97% of the original studies produced statistically significant results, only 36% of the replication studies did so (Open Science Collaboration, 2015). This inability to replicate previously published results, however, is not limited to psychology (Ioannidis, 2005). Replication projects in medicine (Prinz et al., 2011) and behavioral economics (Camerer et al., 2016) resulted in replication rates of 25 and 61%, respectively, and analyses in genetics (Munafò, 2009) and neuroscience (Button et al., 2013) question the validity of studies in those fields. Science, in general, is reckoning with challenges in one of its basic tenets: replication.

Comparative psychology also faces the grand challenge of producing replicable research. Though social psychology has born the brunt of most of the critique regarding failed replications, comparative psychology suffers from some of the same problems faced by social psychology (e.g., small sample sizes). Yet, comparative psychology follows the methods of cognitive psychology by often using within-subjects designs, which may buffer it from replicability problems (Open Science Collaboration, 2015). In this Grand Challenge article, I explore the shared and unique challenges of and potential solutions for replication and reproducibility in comparative psychology.

## 1. Replicability and reproducibility: definitions and challenges

Researchers often use the terms replicability and reproducability interchangeably, but it is useful to distinguish between them. *Replicability* is “re-performing the experiment and collecting new data,” whereas *reproducibility* is “re-performing the same analysis with the same code using a different analyst” (Patil et al., 2016). Therefore, one can replicate a study or an effect (outcome of a study) but reproduce results (data analyses). Each of these three efforts face their own challenges.

### 1.1. Replicating studies

Though science depends on replication, replication studies are rather rare due to an emphasis on novelty: journal editors and reviewers value replication studies less than original research (Neuliep and Crandall, 1990, 1993). This culture is changing with funding agencies (Collins and Tabak, 2014) and publishers (Association for Psychological Science, 2013; McNutt, 2014) adopting policies that encourage replications and reproducible research. The recent wave of replications, however, has resulted in a backlash, with replicators labeled as bullies, ill-intentioned, and unoriginal (Bartlett, 2014; Bohannon, 2014). Much of this has played out in opinion pieces, blogs, social media, and comment sections, leading some to allege a culture of “shaming” and “methodological intimidation” (Fiske, 2016). Nevertheless, replication studies are becoming more common, with some journals specifically soliciting them (e.g., *Animal Behavior and Cognition, Perspectives on Psychological Science*).

### 1.2. Replicating effects

When studies are replicated, the outcomes do not always match the original studies' outcomes. This may result from differences in design and methods between the original and replication studies or from a false negative in the replication (Open Science Collaboration, 2015). However, it also may occur because the original study was a false positive; that is, the original result was spurious. Unfortunately, biases in how researchers decide on experimental design, data analysis, and publication can produce results that fail to replicate. At many steps in the scientific process, researchers can fall prey to confirmation bias (Wason, 1960; Nickerson, 1998) by focusing on positive confirmations of hypotheses. At the experimental design stage, researchers may develop tests that attempt to confirm rather than disconfirm hypotheses (Sohn, 1993). This typically relies on null hypothesis significance testing, which is frequently misunderstood and misapplied by researchers (Nickerson, 2000; Wagenmakers, 2007) and focuses on a null hypothesis rather than alternative hypotheses. At the data collection phase, researchers may perceive behavior in a way that aligns with their expectations rather than the actual outcomes (Marsh and Hanlon, 2007). When analyzing data, researchers may report results that confirm their hypotheses while ignoring disconfirming results. This “p-hacking” (Simmons et al., 2011; Simonsohn et al., 2014) generates an over-reporting of results with *p*-values just under 0.05 (Masicampo and Lalande, 2012) and is a particular problem for psychology (Head et al., 2015). Finally, after data are analyzed, studies with negative or disconfirming results may not get published, causing the “file drawer problem” (Rosenthal, 1979). This effect could also result in under-reporting of replication studies when they fail to find the same effects as the original studies.

### 1.3. Reproducing results

“An article […] in a scientific publication is **not** the scholarship itself, it is merely **advertising** of the scholarship. The actual scholarship is the complete software development environment and the complete set of instructions which generated the figures” (Buckheit and Donoho, 1995, p. 59, emphasis in the original). There are many steps between collecting data and generating statistics and figures reported in a publication. For research to be truly reproducible, researchers must open that entire process to scrutiny. Currently, this is not possible for most publications because the relevant information is not readily accessible to other scientists. For example, in a survey of 441 biomedical articles from 2000 to 2014, only one was fully reproducible (Iqbal et al., 2016). When data and the code generating analyses are unavailable, this prevents truly reproducible research.

## 2. Unique challenges for comparative psychology

In addition to the general factors contributing to the replication crisis across science, working with non-human animals poses unique challenges for comparative psychology.

**Figure 1:**
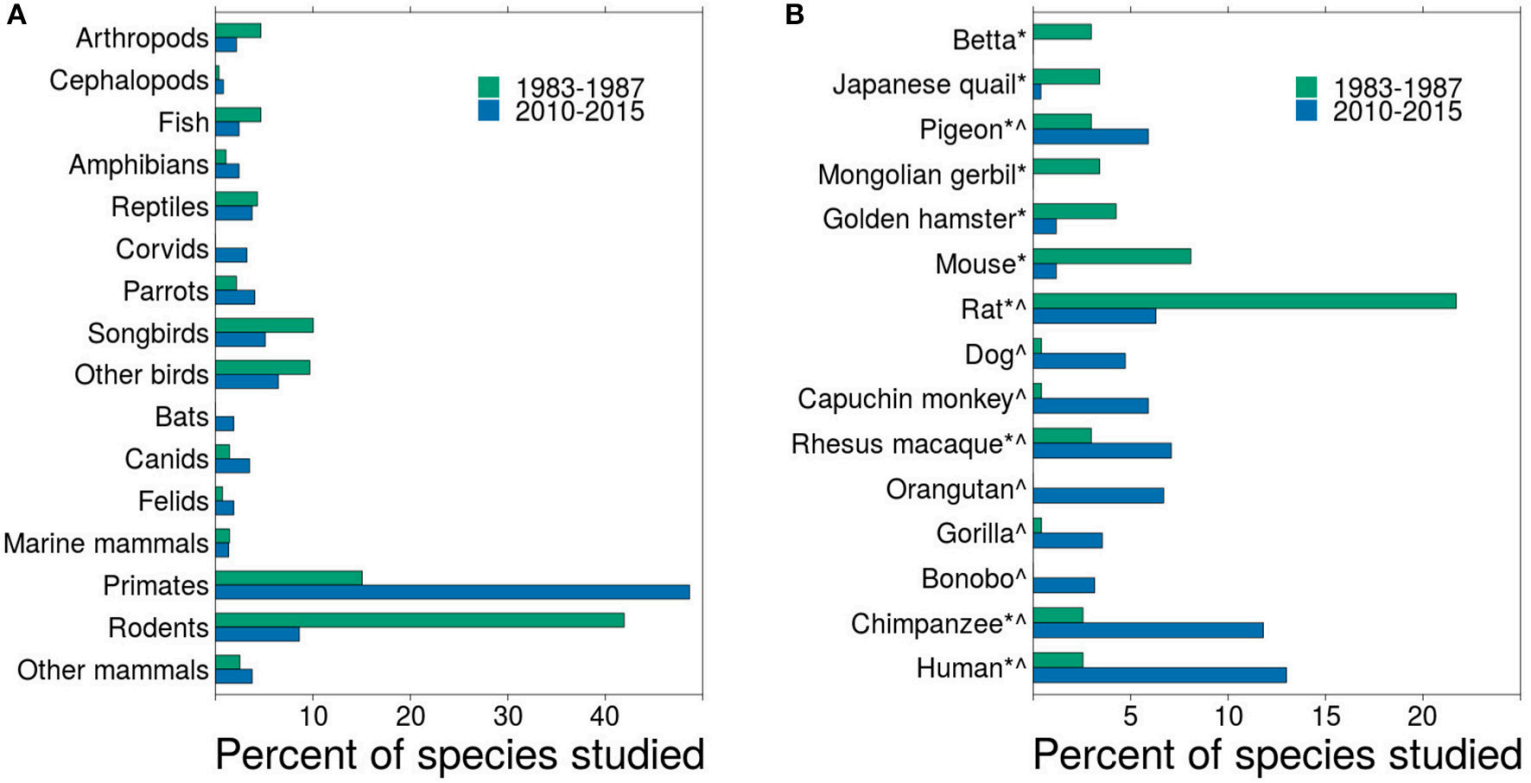
**Changes in species studied in *Journal of Comparative Psychology* from 1983 to 1987 and from 2010 to 2015. (A)** shows the frequency of taxonomic groups included in empirical articles for both time periods. Because the time periods differed in the number of articles published, the percent of species included in all articles is presented. **(B)** shows a subset of the data that includes the top 10 most frequently studied species for each time period. ^*^Top 10 most frequent from 1983 to 1987. ^∧^Top 10 most frequent from 2010 to 2015.

- *Small sample sizes*—With over 7 billion humans on the planet, many areas of psychology have a large population to draw from for research participants. Indeed, given that undergraduate students comprise most of the psychology study participants (Arnett, 2008) and that most colleges and universities have hundreds to tens of thousands of students, recruiting psychology study subjects is relatively easy. Yet, for comparative psychologists, large sample sizes can prove more challenging to acquire due to low numbers of individual animals in captivity, regulations limiting research, and the expense of maintaining colonies of animals. These small sample sizes can prove problematic, potentially resulting in spurious results (Agrillo and Petrazzini, 2012).
- *Repeated testing*—For researchers studying human psychology, colleges and universities refresh the subject pool with a cohort of new students every year. The effort, expense, and logistics of acquiring new animals for a comparative psychology lab, however, can be prohibitive. Therefore, for many labs working with long-lived species, such as parrots, corvids, and primates, researchers test the same individuals repeatedly. Repeated testing can result in previous experimental histories influencing behavioral performance, which can impact the ability of other researchers to replicate results based on these individuals.
- *Exploratory data analysis*—Having few individuals may also drive researchers to extract as much data as possible from subjects. Collecting large amounts of data and conducting extensive analysis on those data is not problematic itself. However, if these analyses are conducted without sufficient forethought and a priori predictions (Anderson et al., 2001; Wagenmakers et al., 2012), exploratory analyses can result in “data-fishing expeditions” (Bem, 2000; Wagenmakers et al., 2011) that produce false positives.
- *Species coverage*—Historically, comparative psychology has focused on a few species, primarily pigeons, mice, and rats (Beach, 1950). Though these species are still the workhorses of the discipline, comparative psychology has enjoyed a robust broadening of the pool of species studied (Shettleworth, 2009, Figure 1). Beach (1950) showed that, in *Journal of Comparative Psychology*, researchers studied about 14 different species a year from 1921 to 1946. From 1983 to 1987, the same journal published studies on 102 different species and, from 2010 to 2015, 144 different species^1^ (Figure 1).
  The increase in species studied is clearly advantageous to the field because it expands the scope of our understanding across a wide range of taxa. But it also has the disadvantage of reducing the depth of coverage for each species, and depth is required for replication. In *Journal of Comparative Psychology* from 1983 to 1987, researchers published 2.3 articles per species. By 2010-2015, that number dropped to 1.8 articles per species. From 1983 to 1987, 62 species were studied in a single article during that 5-year span, whereas from 2010 to 2015, 103 species were studied only in a single article. Some of the species tested are quite rare, which can limit access to them. For example, Gartner et al. (2014) explored personality in clouded leopards, which have fewer than 10,000 individuals in the wild (Grassman et al., 2016) and fewer than 200 in captivity (Wildlife Institute of India, 2014). Expanding comparative psychology to a wide range of species spreads out resources, making replication less likely.
- *Substituting species*—When attempting to replicate or challenge another study's findings, comparative psychologists sometimes turn to the most convenient species to test rather than testing the species used in the original study. This is problematic because substituting a different species is not a direct replication (Schmidt, 2009; Makel et al., 2012), and it is not clear what a failure to replicate or an alternative outcome means across species. Even within a species, strains of mice and rats, for instance, vary greatly in their behavior. Thus, for comparative psychology, a direct replication requires testing the same species and/or strain to match the original study as closely as possible.

## 3. Resolving the crisis

The replicability crisis in psychology has spawned a number of solutions to the problems (Wagenmakers, 2007; Frank and Saxe, 2012; Koole and Lakens, 2012; Nosek et al., 2012; Wagenmakers et al., 2012; Asendorpf et al., 2013). In addition to encouraging more direct replications, these solutions address the problems of null hypothesis significance testing, p-hacking, and reproducing analyses.

### 3.1. Null hypothesis significance testing

- *Effect sizes*—Despite decades of warnings about the perils of null hypothesis significance testing (Rozeboom, 1960; Gigerenzer, 1998; Marewski and Olsson, 2009), psychology has been slow to move away from this tradition. However, a number of publishers in psychology have begun requiring or strongly urging authors to include effect sizes in their statistical analyses. This diverts focus from the binary notion of “significant” or “not significant” to a description of the strength of effects.
- *Bayesian inference*—Another recent trend is to abandon significance testing altogether and switch to Bayesian statistics. While significance testing yields the probability of the data given a hypothesis (Cohen, 1990), a Bayesian approach provides the probability of a hypothesis given the data, which is what researchers typically seek (Wagenmakers, 2007). A key advantage of this approach is that it offers the strength of evidence favoring one hypothesis over another.
- *Multiple hypotheses*—Rather than testing a single hypothesis against a null, researchers can make stronger inferences by developing and testing multiple hypotheses (Chamberlin, 1890; Platt, 1964). Information-theoretic approaches (Burnham and Anderson, 2010) and Bayesian inference (Wagenmakers, 2007) allow researchers to test the strength of evidence among these hypotheses.

### 3.2. P-hacking

- *Labeling confirmatory and exploratory analyses*—Confirmatory data analysis tests a priori hypotheses. Analyzing data after observing the data, however, is exploratory analysis. Though exploratory analysis is not inherently ‘bad’, it is disingenuous and statistically invalid to treat exploratory analyses as confirmatory analyses (Wagenmakers et al., 2011, 2012). To clarify between these types of analyses, researchers should clearly label confirmatory and exploratory analyses. Also, researchers can convert exploratory analyses to confirmatory analyses by collecting follow-up data to replicate the exploratory effects.
- *Pre-registration*—A more rigorous way for researchers to avoid p-hacking and data-fishing expeditions is to commit to specific data analyses before collecting data. Researchers can pre-register their studies at pre-registration websites (e.g., https://aspredicted.org/) by specifying in advance the research questions, variables, experimental methods, and planned analyses (Wagenmakers et al., 2012). Registered reports take this a step forward by subjecting the pre-registration to peer review. Journals that allow registered reports agree that “manuscripts that survive pre-study peer review receive an in-principle acceptance that will not be revoked based on the outcomes”, though they may be rejected for other reasons (Center for Open Science, 2016).

### 3.3. Reproducing analyses

- *Archiving data and analyses*—A first step toward reproducing data analysis is to archive the data (and a description of it) publicly, which allows other researchers to access the data for their own analyses. An important second step is to archive a record of the analysis itself. Many software packages allow researchers to output the scripts that generate the analyses. The statistical software package R (R Core Team, 2017) is free, publicly available software that allows researchers to save scripts of the statistical analysis. Archiving the data and R scripts makes the complete data analysis reproducible by anyone without requiring costly software licenses. Data repositories, such as Dryad (http://datadryad.org/) and Open Science Framework (https://osf.io/) archive these files.
- *Publishing workflows*—The process from developing a research question to submitting a manuscript for publication takes many steps and long periods of time, usually on the order of years. A perfectly reproducible scientific workflow would track each step of this process and make them available for others to access (Nosek et al., 2012). Websites, such as the Open Science Framework (https://osf.io/) can manage scientific workflows for projects by providing researchers a place to store literature, IRB materials, experimental materials (stimuli, software scripts), data, analysis scripts, presentations, and manuscripts. This workflow management system allows researchers to collaborate remotely and make the materials publicly available for other researchers to access.

### 3.4. Replicability in comparative psychology

Comparative psychologists can improve the rigor and replicability by following these general recommendations. However, a number of practices specific to the field will improve our scientific rigor.

- *Multi-species studies*—Though many comparative psychology studies have smaller samples sizes than is ideal, testing multiple species in a study can boost sample size. *Journal of Comparative Psychology* showed an increase in the number of species tested per article from 1.2 in 1983–1987 to 1.5 in 2010–2015. Many of these studies explore species differences in learning and cognition, but they can also act as replications across species.
- *Multi-lab collaborations*—To investigate the replication problem in human psychology, researchers replicate studies across different labs (Klein et al., 2014; Open Science Collaboration, 2015). Multi-lab collaborations are more challenging for comparative psychologists because there is a limited number of other facilities with access to the species under investigation. Nevertheless, comparative psychologists do engage in collaborations testing the same species in different facilities (e.g., Addessi et al., 2013). A more recent research strategy is to conduct the same experimental method across a broad range of species in different facilities (e.g., MacLean et al., 2014). Again, though these studies are often investigating species differences, showing similarities across species acts as a replication.
- *Accessible species*—Captive animal research facilities are increasingly under pressure due to increased costs and changing regulations and funding priorities. In the last decade, many animal research facilities have closed, and researchers have turned to more accessible species, especially dogs. Because researchers do not have to house people's pets, the costs of conducting research on dogs is much lower than maintaining an animal facility. A key advantage of dog research is that they are abundant, with about 500 million individuals worldwide (Coren, 2012). This allows ample opportunities for large sample sizes. Moreover, researchers are opening dog cognition labs all over the world, which provides the possibility of multi-lab collaborations and replications.
- *Accessible facilities*—Another alternative to maintaining animal research facilities is to leverage existing animal colonies. Zoos provide a wide variety of species to study and are available in many metropolitan areas. Though the sample sizes per species may be low, collaboration across zoos is possible. Animal sanctuaries provide another avenue for studying more exotic species, potentially with large sample sizes.

In summary, like all of psychology and science in general, comparative psychology can improve its scientific rigor by rewarding and facilitating replication, strengthening statistical methods, avoiding p-hacking, and ensuring that our methods, data, and analyses are reproducible. In addition, comparative psychologists can use field-specific strategies, such as testing multiple species, collaborating across labs, and using accessible species and facilities to improve replicability.

*Frontiers in Comparative Psychology* will continue to publish high-quality research exploring the psychological mechanisms underlying animal behavior (Stevens, 2010). To help meet the grand challenge of replicability and reproducability in comparative psychology, I highly encourage authors to (1) conduct and submit replications of their own or other researchers' studies, (2) participate in cross-lab collaborations, (3) pre-register methods and data analysis, (4) use robust statistical methods, (5) clearly delineate confirmatory and exploratory analyses/results, and (6) publish data and statistical scripts with their research articles and on data repositories. Combining these solutions can ensure the validity of our science and the importance of animal research for the future.

## Author contributions

The author confirms being the sole contributor of this work and approves it for publication.

### Conflict of interest statement

The author declares that the research was conducted in the absence of any commercial or financial relationships that could be construed as a potential conflict of interest.

## Footnotes

^1^I downloaded from Web of Science citation information from all articles published in *Journal of Comparative Psychology* (*JCP*) from 1983 (when *Journal of Comparative and Physiological Psychology* separated into *JCP* and *Behavioral Neuroscience*) to 1987 (*N* = 235) and from 2010 to 2015 (*N* = 254). Based on the title and abstract, I coded the species tested in all empirical papers. Data and the R script used to analyze the data are available as supplementary materials.
