# Supplementary material for: Replicability and Reproducibility in Comparative Psychology
Source: Front Psychol. 2017 May 26;8:862. doi: 10.3389/fpsyg.2017.00862 (PMC5445189; doi:10.3389/fpsyg.2017.00862)
Supplement: Supplementary file 1 [file RScript.PDF]

```r
###################################################
### Created by Jeffrey R. Stevens on 20 Dec 2016 (jeffrey.r.stevens@gmail.com),
###   finalized on 9 Jan 2017
### Summary: This script loads and prepares the Journal of Comparative Psychology
###   data sets and generates figures.
### Instructions: Source this file (stevens_rcode.R) to run the script and output figures.
### Uses: This script can be reproduced and modified for personal and scientific use.
### Description of the columns for stevens_data.csv:
###   commmon_name - species common name
###   speices - species scientific name
###   group - species taxanomic group
###   year - five-year period of analysis
###################################################

rm(list = ls())   # clear all variables

##########################
## Load libraries
##########################
library(car)      # needed for Recode
library(lattice)  # needed for lattice barcharts

##########################
## Load and prepare data
##########################
data <- read.csv("data/stevens_data.csv") # input data file
data$taxa <- Recode(data$group, "'Arachnid'='Arthropod';'Crustacean'='Arthropod';'Insect'='Arthropod'") # recode taxanomic groups
eighties_articles <- 235  # assign number of articles analyzed from 1983-1987
recent_articles <- 254    # assign number of articles analyzed from 2010-2015
col.blind <- c("#0072B2", "#E69F00", "#009E73", "#D55E00")    # prepare color-blind-safe colors

eighties <- subset(data, year == "1983-1987") # subset data from 1983-1987
recent <- subset(data, year == "2010-2015")   # subset data from 2010-2015

##########################
## Calculate relative frequencies of species
##########################
## For 1983-1987 data
eighties_table_all <- table(eighties$species)                           # count number of each species studied
eighties_table <- eighties_table_all[eighties_table_all > 0]            # subset species with at least one study
eighties_species <- length(eighties_table)                              # calculate the total number of species studies
eighties_articles_per_species <- eighties_articles / eighties_species   # calculate number of articles per species studies
eighties_one_study_species <- sum(eighties_table[eighties_table == 1])  # calculate number of species studied once
eighties_taxa <- table(eighties$taxa) / sum(table(eighties$taxa)) * 100 # calculate percent of species studied per taxa

## For 2010-2015 data
recent_table_all <- table(recent$species)                         # count number of each species studied
recent_table <- recent_table_all[recent_table_all > 0]            # subset species with at least one study
recent_species <- length(recent_table)                            # calculate the total number of species studies
recent_articles_per_species <- recent_articles / recent_species   # calculate number of articles per species studied
recent_one_study_species <- sum(recent_table[recent_table == 1])  # calculate number of species studied once
recent_taxa <- table(recent$taxa) / sum(table(recent$taxa)) *100  # calculate percent of species studied per taxa

##########################
## All taxa analyses
##########################
eighties_taxa_df <- data.frame(eighties_taxa)                             # create data frame
recent_taxa_df <- data.frame(recent_taxa)                                 # create data frame
names(eighties_taxa_df) <- names(recent_taxa_df) <- c("taxa", "count")    # rename columns
eighties_taxa_df$time <- rep("1983-1987", length(eighties_taxa_df[, 1]))  # create column of time period
recent_taxa_df$time <- rep("2010-2015", length(recent_taxa_df[, 1]))      # create column of time period
```

```
combined_taxa_df <- rbind(eighties_taxa_df, recent_taxa_df)                # combine data from both time periods
combined_taxa_df$taxa <- factor(combined_taxa_df$taxa, labels = c("Amphibians", "Arthropods", "Bats", "Other birds", "Canids", "Cephalopods", "Corvids",
"Felids", "Fish", "Other mammals", "Marine mammals", "Parrots", "Primates", "Reptiles",  "Rodents", "Songbirds")) # reorder taxa
combined_taxa_df$taxa <- factor(combined_taxa_df$taxa, levels = c("Other mammals", "Rodents", "Primates", "Marine mammals", "Felids", "Canids", "Bats",
"Other birds", "Songbirds", "Parrots", "Corvids", "Reptiles", "Amphibians", "Fish", "Cephalopods", "Arthropods")) # rename taxa
combined_taxa_df$time <- as.factor(combined_taxa_df$time) # convert time periods to factors
combined_taxa_df$time <- factor(combined_taxa_df$time, levels = c("2010-2015", "1983-1987"))  # reorder time periods

# Plot barchart of taxa frequencies
taxa_plot <- barchart(taxa ~ count, groups = time, combined_taxa_df,
  aspect = 1.4, col = col.blind[c(1, 3)],
  xlab = "Percent of species studied", ylab = "", xlim = c(0, 50),
  par.settings = list(axis.text = list(cex = 2.25), par.xlab.text = list(cex = 3.5), par.ylab.text = list(cex = 3.5)),
  key = list(corner = c(0.85, 0.9), points = list(pch = 15, cex = 3.5, col = col.blind[c(3, 1)]), text = list(levels(combined_taxa_df$time)[2:1]), type =
"p", divide = 1, cex = 2.25)
)
png(filename = "stevens_fig1a.png", width = 800, height = 800) # create PNG device
plot(taxa_plot)  # plot figure
dev.off() # close device

##########################
## Species analyses
##########################
eighties_percent_all <- eighties_table_all / eighties_articles * 100        # calculate percent of species studied
recent_percent_all <- recent_table_all / recent_articles * 100              # calculate percent of species studied
eighties_df <- data.frame(eighties_table_all, eighties_percent_all)         # create data frame
eighties_df <- eighties_df[-3]                                              # remove redundant species column
recent_df <- data.frame(recent_table_all, recent_percent_all)               # create data frame
recent_df <- recent_df[-3]                                                  # remove redundant species column
names(eighties_df) <- names(recent_df) <- c("species", "count", "percent")  # rename columns
eighties_df$time <- rep("1983-1987", length(eighties_df[, 1]))              # create column of time period
recent_df$time <- rep("2010-2015", length(recent_df[, 1]))                  # create column of time period
combined_df <- rbind(eighties_df, recent_df)                                # combine data from both time periods
eighties_df <- eighties_df[order(eighties_df$count, decreasing = TRUE), ]   # reorder data frame by frequency count
recent_df <- recent_df[order(recent_df$count, decreasing = TRUE), ]         # reorder data frame by frequency count
eighties_trimmed_df <- eighties_df[1:10, ]                                  # subset top 10 most frequent species
recent_trimmed_df <- recent_df[1:10, ]                                      # subset top 10 most frequent species
combined_trimmed_df_wide <- merge(eighties_trimmed_df, recent_trimmed_df, by = "species", all = TRUE) # merge top 10 most frequent species for both time
periods
combined_trimmed_df <- subset(combined_df, species %in% unique(combined_trimmed_df_wide$species)) # subset all data both species in combined top 10 lists
combined_trimmed_df$species <- factor(combined_trimmed_df$species, levels = c("Homo sapiens", "Pan troglodytes", "Pan paniscus","Gorilla gorilla","Pongo
spp.", "Macaca mulatta", "Sapajus apella", "Canis familiaris", "Rattus norvegicus", "Mus musculus", "Mesocricetus auratus", "Meriones unguiculatus",
"Columba livia", "Coturnix japonica", "Betta splendens")) # reorder species
combined_trimmed_df$species <- factor(combined_trimmed_df$species, labels = c("Human*^", "Chimpanzee*^", "Bonobo^", "Gorilla^", "Orangutan^", "Rhesus
macaque*^", "Capuchin monkey^", "Dog^", "Rat*^", " Mouse*", "Golden hamster*", "Mongolian gerbil*", "Pigeon*^", "Japanese quail*", "Betta*"))  # rename
species (* = top 10 1983-1987, ^ = top 10 2010-2015)
combined_trimmed_df$time <- factor(combined_trimmed_df$time, levels = c("2010-2015", "1983-1987"))  # reorder time periods

# Plot barchart of species frequencies
species_plot <- barchart(species ~ percent, groups = time, combined_trimmed_df,
  aspect = 1.4, col = col.blind[c(1, 3)],
  xlab = "Percent of species studied", ylab = "", xlim = c(0, 25),
  par.settings = list(axis.text = list(cex = 2.25), par.xlab.text = list(cex = 3.5), par.ylab.text = list(cex = 3.5)),
  key = list(corner = c(0.85, 0.9), points = list(pch = 15, cex = 3.5, col = col.blind[c(3, 1)]), text = list(levels(combined_trimmed_df$time)[2:1]),
type = "p", divide = 1, cex = 2.25)
)
png(filename = "stevens_fig1b.png", width = 800, height = 800) # create PNG device
plot(species_plot)  # plot figure
dev.off() # close device
```
